# Spanning Trees of Lattices Embedded on the Klein Bottle

**DOI:** 10.1155/2014/452453

**Published:** 2014-08-27

**Authors:** Fuliang Lu

**Affiliations:** School of Sciences, Linyi University, Linyi, Shandong 276000, China

## Abstract

The problem of enumerating spanning trees in lattices with Klein bottle boundary condition is considered here. The exact closed-form expressions of the numbers of spanning trees for 4.8.8 lattice, hexagonal lattice, and 3^3^ *·*4^2^ lattice on the Klein bottle are presented.

## 1. Introduction 

Let *G* = (*V*(*G*), *E*(*G*)) denote a graph with no multiple edges and no loops and with vertex set *V*(*G*) = {*v*_1_, *v*_2_,…, *v*_*n*_} and edge set *E*(*G*). The degree *k*_*s*_ of a vertex *v*_*s*_ is the number of edges attached to it. A *k*-regular graph is a graph with the property that each of its vertices has the same degree *k*. The adjacency matrix *A*(*G*) of *G* is the *n* × *n* matrix with elements *A*(*G*)_*sj*_ = 1 if *v*_*s*_ and *v*_*j*_ are connected by an edge and zero otherwise. The Laplacian matrix *Q*(*G*) is the *n* × *n* matrix with the element *Q*(*G*)_*sj*_ = *k*_*s*_ *δ*_*sj*_ − *A*(*G*)_*sj*_, where *δ*_*sj*_ is the Kronecker delta, equal to 1 if *s* = *j*, and zero otherwise. Denote by *t*(*G*) the number of spanning trees of a graph *G*. Enumeration of spanning trees on the graph is a problem of fundamental interest in mathematics and physics. This number can be calculated in several ways. A basic result is “the Matrix-Tree Theorem.”

**Theorem 1 (see [1]).**

Let *G* be a graph with vertex set {*v*_1_, *v*_2_,…, *v*_*n*_} and let *Q*(*G*) be its Laplacian matrix. Then,

(1) $$\begin{array}{c} t\left(G\right)=\det\left(Q{\left(G\right)}^{\left\{s\right\}}\right), \end{array}$$

where *Q*(*G*)^{*s*}^ is the submatrix of *Q*(*G*) by deleting the sth row and the sth column from *Q*(*G*) for 1 ≤ *s* ≤ *n*.

Note that one of the eigenvalues of *Q*(*G*) is always zero. We can express *t*(*G*) that can be expressed by the nonzero eigenvalue of *Q*(*G*) as follows.

**Lemma 2 (see [1]).**

Let 0 < *μ*_1_ ≤ *μ*_2_ ≤ ⋯≤*μ*_*n*−1_ be the Laplacian eigenvalues of a connected graph *G* with *n* vertices. Then, *t*(*G*) = *μ*_1_ *μ*_2_ … *μ*_*n*−1_/*n*.

By two methods, Ciucu et al. [2] obtained a factorization theorem for the number of spanning trees of the plane graphs with reflective symmetry (all orbits have two vertices). In [3], Zhang and Yan obtained a factorization theorem for the number of spanning trees of the more general graphs with reflective symmetry (i.e., the so-called graphs with an involution, and all orbits have one or two vertices). A graph *G* is said to be *n*-rotational symmetric if the cyclic group of order *n* is a subgroup of the automorphism group of *G*. Yan and Zhang [4] also obtained a factorization theorem for *n*-rotational symmetric graph. As applications, they got explicit expressions for the numbers of spanning trees and the asymptotic tree number entropy for some lattices with cylindrical boundary condition.

Lattices are of special interest for their structures. In particular, the number of spanning trees in a lattice was studied extensively. It turns out that *t*(*G*) has asymptotically exponential growth; one defines the quantity *z*(*G*) by

(2) $$\begin{array}{c} z\left(G\right)=\underset{|V(G)|\to \infty }{\lim}\frac{\log t\left(G\right)}{\left|V\left(G\right)\right|}. \end{array}$$

This limit is known as the asymptotic tree number entropy, asymptotic growth constant, or thermodynamical limit.

Closed-form expressions for *t*(*G*) have been obtained for many lattices. Wu [5] evaluated the number of spanning trees on a large planar lattice, exactly for the square, triangular, and honeycomb lattice. Tzeng and Wu [6] obtained the spanning tree generating function for a hypercubic lattice in *d* dimensions under free, periodic, and a combination of free and periodic boundary conditions and a quartic lattice embedded on a Möbius strip and the Klein bottle. Shrock and Wu [7] got a general formulation for the number of spanning trees on lattices in high dimensions. With the formulation, closed-form expressions for the number of spanning trees for hypercubic, body-centred cubic, face-centred cubic, and specific planar lattices including the kagomé, diced, 4.8.8 (bathroom-tile), Union Jack, and 3.12.12 lattices are obtained. With the same method, Chang and Shrock [8] got closed-form expressions of the number of spanning trees for the *d*-dimensional body-centred cubic lattice and thermodynamical limit. They also gave an exact integral expression for thermodynamical limit on the face-centred cubic lattice and 4.8.8 lattice. Chang and Wang [9] considered the number of spanning trees of some Archimedean lattices and hypercubic lattices. More related results can be found in [10, 11].

In this paper, we present an exact closed-form result for the asymptotic growth constant for spanning trees of lattices embedded on Klein bottle, exactly for 4.8.8 lattice, hexagonal lattice, and 3^3^ · 4^2^ lattice. The number of spanning trees of 4.8.8 lattice is gotten in Section 2. With the same method, we consider hexagonal lattice and 3^3^ · 4^2^ lattice in Sections 3 and 4, respectively.

## 2. The 4.8.8 Lattice

Introduce some notation firstly. Let *B* ^−1^ and *B* ^*T*^ be the inverse and the transpose of a matrix *B*. And let *I*_*m*_ denote the *m* × *m* identity matrix. Set

(3) Rn=[010⋯00010⋯⋮⋮⋱⋱⋱00⋯0100⋯00]n×n,K1=[010⋯00010⋱⋮⋮⋮⋱⋱000⋯1100⋯0]m×m,K2=[11⋰1]m×m.

Let *U* be an *m* × *m* matrices with entries

(4) $$\begin{array}{c} U_{t,j}=\sqrt{\frac{1}{m}}e^{i(2jt\pi /m)};\;t,j=1,2,\ldots ,m. \end{array}$$

It is not difficult to check that the elements of the *m* × *m* matrices *U* ^−1^ are

(5) $$\begin{array}{c} {\left(U^{-1}\right)}_{t,j}=\sqrt{\frac{1}{m}}e^{-i(2tj\pi /m)}. \end{array}$$

The entries of the *m* × *m* matrices *U* ^−1^ *K*_1_ *U*, *U* ^−1^(*K*_1_^*T*^)*U* and *U* ^−1^ *K*_2_ *U* are

(6) (U−1K1U)t,j=eiθtδt,j,  (U−1(K1T)U)t,j=e−iθtδt,j,(U−1K2U)t,j=e−iθtδt+j,n,

where *θ*_*t*_ = 2*tπ*/*m* for *t*, *j* = 1,2,…, *m*.

**Figure 1:**
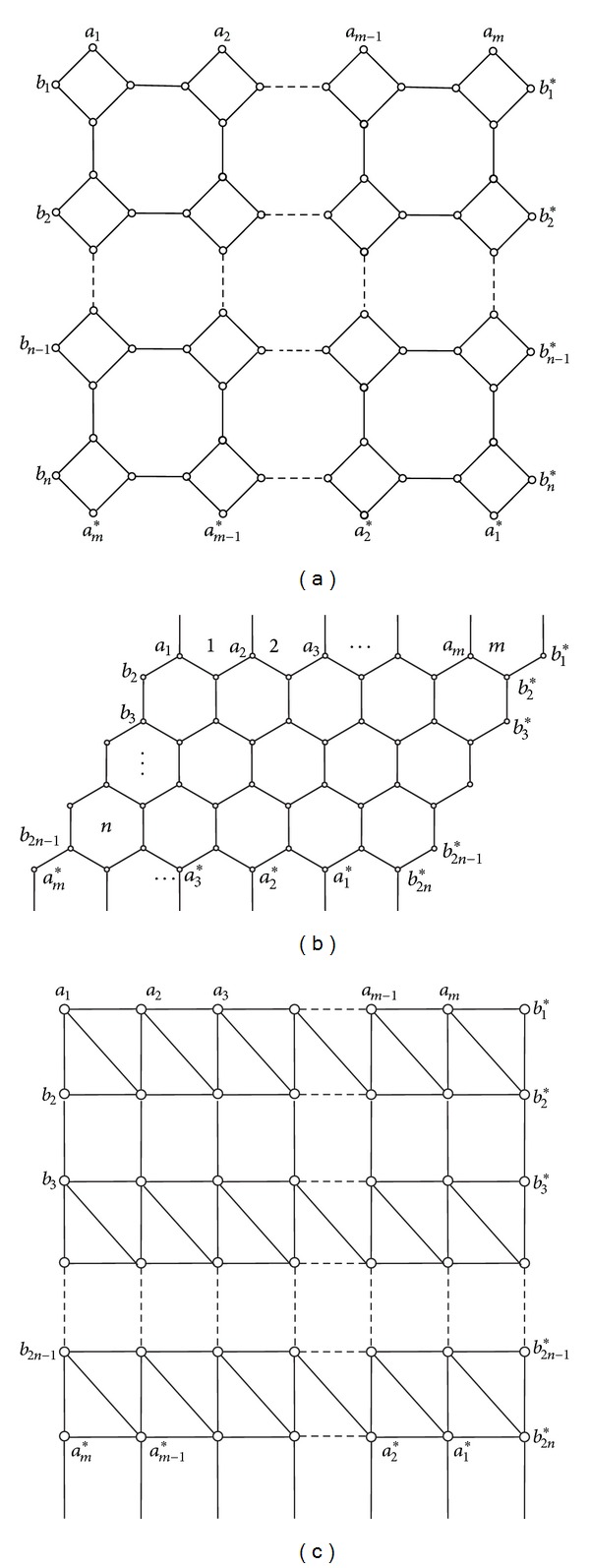
(a) The 4.8.8 lattice; (b) the hexagonal lattice; (c) the 3^3^ · 4^2^ lattice.

The 4.8.8 lattice *L*_4.8.8_ is shown in Figure 1(a). If we add edges (*b*_*j*_, *b*_j_*), for 1 ≤ *j* ≤ *n* in *L*_4.8.8_, we obtain a graph with cylindrical boundary condition, denoted by *L*_4.8.8_^*c*^. Adding edges (*a*_*s*_, *a*_*m*+1−s_*), for 1 ≤ *s* ≤ *m* in *L*_4.8.8_^*c*^, a 4.8.8 lattice with toroidal boundary condition, denoted by *L*_4.8.8_^*t*^, can be gotten.

Yan and Zhang [4] got the number of spanning trees and the asymptotic tree number entropy of *L*_4.8.8_^*c*^:

(7) t(L4.8.8c)=8nm∏j=1m−1ab[(c+4ab)n−(c−4ab)n],z(L4.8.8c)=lim⁡m,n→∞14mnlog⁡t(L4.8.8c)=14log⁡2+14π ×∫0πlog⁡⁡[7−3cos⁡x+4sin⁡(x2)5−cos⁡x]dx≈0.7867,

where *a* = 1 − cos⁡(2*jπ*/*m*), *b* = 10 − 2cos⁡(2*jπ*/*m*), and *c* = 14 − 6cos⁡(2*jπ*/*m*).

Shrock and Wu [7] showed that the number of spanning trees and the asymptotic tree number entropy of *L*_4.8.8_^*t*^ can be expressed as

(8) t(L4.8.8t)=16nm ×∏s=0n−1 ∏j=0m−1(s,j)≠(0,0)4(7−3cos⁡θ1−3cos⁡θ2−cos⁡θ1cos⁡θ2),z(L4.8.8t)=lim⁡m,n→∞14mnlog⁡t(L4.8.8t)=14log⁡2+14π ×∫0πlog⁡⁡[7−3cos⁡x+4sin⁡(x2)5−cos⁡x]dx,≈0.7867,

where *θ*_1_ = 2*sπ*/*n* and *θ*_2_ = 2*jπ*/*m*. Chang and Shrock [8] obtained a closed-form expression of *L*_4.8.8_^*t*^ by an exact closed-form evaluation of the integral given in [7].

By adding edges (*a*_*s*_, *a*_s_*), for 1 ≤ *s* ≤ *m* in *L*_4.8.8_^*c*^, 4.8.8 lattice *L*_4.8.8_^*K*^ with Klein bottle boundary condition can be gotten. By a suitable labelling of vertices of *L*_4.8.8_^*K*^, the adjacency matrix *X* of it can be written in terms of a linear combination of direct products of smaller ones:

(9) $$\begin{array}{c} X=A\underset{}{⊗}I_{m}+B\underset{}{⊗}K_{1}+B^{T}\underset{}{⊗}K_{1}^{T}+C\underset{}{⊗}K_{2}, \end{array}$$

where

(10) A[0110100110010110]⊗In+[0000000000001000]⊗Rn +[0001000000000000]⊗RnT,B=[0000000001000000]⊗In,  C=[00⋯0100⋯00⋮⋮⋯⋮⋮00⋯0010⋯00]4n×4n.

By (6), we have

(11) (I4n⊗U)−1(dI4mn−X)(I4n⊗U) =(I4n⊗U)−1[(dI4n−A)⊗Im−B⊗K1−BT⊗K1T−C⊗K2](I4n⊗U) =(dI4n−A)⊗(U−1ImU)−B⊗(U−1K1U)−BT⊗(U−1(K1T)U)−C⊗(U−1K2U)={[A1′C1′0A2′C2′⋱⋰⋮Am/2′+Cm/2′⋰⋱⋮Cm−2′Am−2′Cm−1′Am−1′00⋯⋯0Am′+Cm′],if  m  is  even,[A1′C1′0A2′C2′⋱⋰⋮⋰⋱⋮Cm−2′Am−2′Cm−1′Am−1′00⋯⋯0Am′+Cm′],if  m  is  odd,

where for *j* = 1,2,…, *m*,

(12) Aj′=dI4n−A−eiθjB−e−iθjBT=D1(θj)⊗In−D2⊗Rn−(D2)T⊗RnT,Cj′=−e−iθjC,D1(x)=[d−1−10−1d−e−ix−1−1−eixd−10−1−1d],  D2=[0000000000001000],

*d* is the degree of the vertices of *L*_4.8.8_^*K*^.

Interchanging rows and columns, those matrices can be changed into a block-diagonal form having the same determinants:

(13) $$\begin{array}{c} \det\left(X\right)=\{\begin{array}{cc} \left|\begin{array}{cccccc} L_{1} & & & & & \\ & L_{2} & & & & \\ & & \ddots & & & \\ & & & L_{m/2-1} & & \\ & & & & A_{m/2}^{\prime }+C_{m/2}^{\prime } & 0\\ & & & & 0 & A_{m}^{\prime }+C_{m}^{\prime } \end{array}\right|, & \\ \;\;\text{if}\;m\;\text{is}\;\text{even}, & \\ \left|\begin{array}{ccccc} L_{1} & & & & \\ & L_{2} & & & \\ & & \ddots & & \\ & & & L_{(m-1)/2} & \\ & & & & A_{m}^{\prime }+C_{m}^{\prime } \end{array}\right|, & \\ \;\;\text{if}\;m\;\text{is}\;\text{odd}, & \end{array} \end{array}$$

where $L_{j}=\left[\begin{array}{cc} A_{j}^{\prime } & C_{j}^{\prime }\\ C_{m-j}^{\prime } & A_{m-j}^{\prime } \end{array}\right]$.

For an even value of *m* (the case when *m* is odd is similar), the Laplacian characteristic polynomial of *L*_4.8.8_^*K*^ can be expressed as

(14) ϕ(L4.8.8K,x)=det⁡⁡(xI4mn−(dI4mn−X))=ϕ1(x)ϕ2(x)⋯ϕm/2(x)ϕm(x),

where

(15) $$\begin{array}{c} \phi_{j}\left(x\right)=\det\left(xI_{8n}-\left[\begin{array}{cc} A_{j}^{\prime } & C_{j}^{\prime }\\ C_{m-j}^{\prime } & A_{m-j}^{\prime } \end{array}\right]\right) \end{array}$$

for *j* = 1,…, *m*/2 − 1, *ϕ*_*j*_(*x*) = det⁡(*xI*_4*n*_ − *A*_*j*_′ − *C*_*j*_′) and for *j* = *m*/2, *m*. Note that

(16) ddxϕ(L4.8.8K,x)=ϕm′(x)∏j=1m/2ϕj(x) +ϕm(x)∑j=1m/2∏k=1m/2ϕk(x)ϕj(x)ϕj′(x).

Hence, by Lemma 2,

(17) 4mnt(L4.8.8K)=μ1μ2⋯μ4mn−1=(−1)4mn−1ddxϕ[L4.8.8K,x]|x=0=−ϕm′(0)∏j=1m/2ϕj(0) −ϕm(0)[∑j=1m/2∏k=1m/2ϕk(x)ϕj(x)ϕj′(x)]|x=0,

where *μ*_1_, *μ*_2_,…, *μ*_4*mn*−1_ are the nonzero Laplacian eigenvalues of *L*_4.8.8_^*K*^.

**Figure 2:**
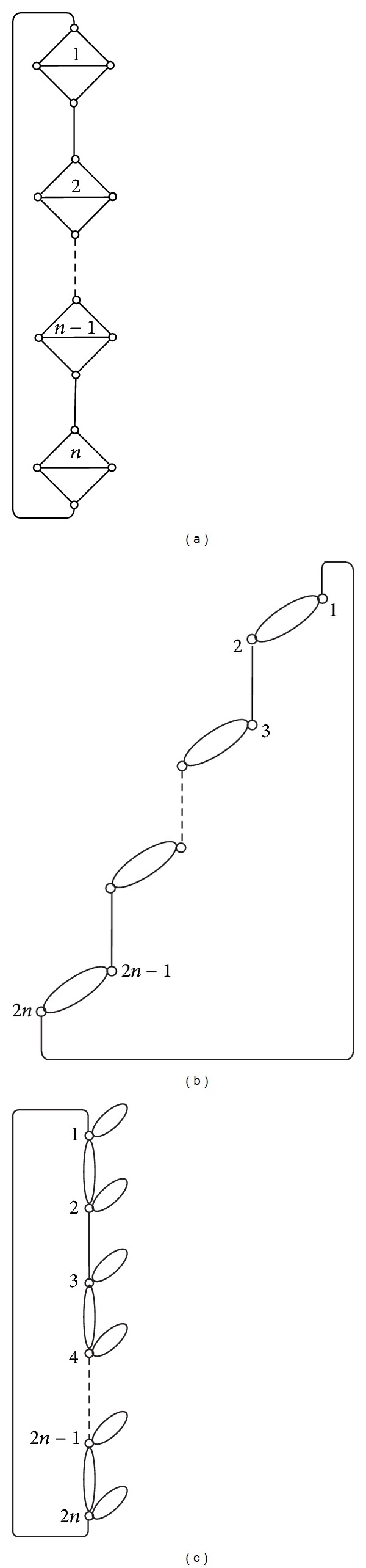
(a) *L*_4.8.8_^0^; (b) *L*_*h*_^0^; (c) *L*_3^3^·4^2^_^0^.

Note that the matrix *dI*_4*n*_ − *A* − *B* − *B* ^*T*^ − *C* also is a Laplacian matrix of a graph, denoted by *L*_4.8.8_^0^ (see Figure 2(a)). Then, *ϕ*_*m*_(0) = det⁡(−*A*_*m*_′ − *C*_*m*_′) = det⁡(−*dI*_4*n*_ + *A* + *B* + *B* ^*T*^ + *C*) = 0 and *ϕ*_*m*_′(0) = (−1)^4*n*−1^4*nt*(*L*_4.8.8_^0^). So, we have

(18) $$\begin{array}{c} mt\left(L_{4.8.8}^{K}\right)=t\left(L_{4.8.8}^{0}\right)\overset{m/2}{\underset{j=1}{\prod }}\phi_{j}\left(0\right). \end{array}$$

Formula (18) is also suitable for other lattices on the Klein bottle with similar proof, but making use of different Laplacian matrix. It will be used later on for two other types of lattices, replacing *L*_4.8.8_^0^ by *L*_*h*_^0^ (see Figure 2(b)) and *L*_3^3^·4^2^_^0^ (see Figure 2(c)), respectively.

In the following, we turn to calculate *ϕ*_*j*_(0). Let *Y* be a subset of the row/column index set of *P*. For convenience, let *P* ^*Y*^ denote the determinant of the matrix obtained from *P* by deleting all rows and columns whose indices are in *Y*. For *j* = 1,…, *m*/2 − 1, noticing that *θ*_*j*_ = −*θ*_*m*−*j*_ and *D*_1_(*θ*_*j*_) = *D*_1_(−*θ*_*j*_), expanding the determinant $\phi_{j}(0)=\det\left[\begin{array}{cc} A_{j}^{\prime } & C_{j}^{\prime }\\ C_{m-j}^{\prime } & {A_{m-j}^{\prime }}_{} \end{array}\right]$, along the first row, and then expanding the resulting determinants along the first column, we have

(19) ϕj(0)=|D1(θj)⊗In−D2⊗R−(D2)T⊗RTe−iθjCe−iθm−jCD1(θm−j)⊗In−D2⊗R−(D2)T⊗RT|=|D1(θj)D2e−iθjD2TD2TD1(θj)D2⋱⋱⋱D2TD1(θj)D2D2TD1(θj)e−iθjD2eiθjD2TD1(−θj)D2D2TD1(−θj)D2⋱⋱⋱D2TD1(−θj)D2eiθjD2D2TD1(−θj)|=3ϕj(0){1}−6(3+cos⁡θj)ϕj(0){1,2,3,4}+(6+2cos⁡θj)ϕj(0){1,2,3,4,5} −ϕj(0){1,8n}−2(6+2cos⁡θj)2n.

Now, we turn to calculate *ϕ*_*j*_(0)^{1}^, *ϕ*_*j*_(0)^{1,2,3,4}^, *ϕ*_*j*_(0)^{1,2,3,4,5}^, and *ϕ*_*j*_(0)^{1,8*n*}^.

Let *F*_2*n*_ = *ϕ*_*j*_(0)^{1}^, *L*_2*n*−1_ = *ϕ*_*j*_(0)^{1,2,3,4}^, *F*_2*n*_′ = *ϕ*_*j*_(0)^{1,8*n*}^, and *L*_2*n*−1_′ = *ϕ*_*j*_(0)^{1,2,3,4,8*n*}^. Also set Γ_*j*−1_ = Γ_*j*_^{1,2,3,4}^, *j* = 2,…, *n*, Γ_*n*_ ∈ {*F*_*n*_, *L*_*n*_, *F*_*n*_′, *L*_*n*_′}.

By the Laplace expansion theorem, we obtain several expansions. First, an expansion by rows 1,2, and 3:

(20) $$\begin{array}{c} F_{n}=\left(18-2\cos\theta_{j}\right)L_{n-1}-8F_{n-1};\\ F_{n}^{\prime }=\left(18-2\cos\theta_{j}\right)L_{n-1}^{\prime }-8F_{n-1}^{\prime }. \end{array}$$

An expansion by rows 1,2, 3, and 4, we get

(21) $$\begin{array}{c} L_{n}=\left(36-12\cos\theta_{j}\right)L_{n-1}-\left(18-2\cos\theta_{j}\right)F_{n-1};\\ L_{n}^{\prime }=\left(36-12\cos\theta_{j}\right)L_{n-1}^{\prime }-\left(18-2\cos\theta_{j}\right)F_{n-1}^{\prime }. \end{array}$$

The recursion relations (20) and (21) give

(22) Γn=(28−12cos⁡θj)Γn−1−(6+2cos⁡θj)Γn−2, Γn∈{Fn,Ln,Fn′,Ln′}.

Note that

(23) F0=0,  F1=det⁡[3−eiθj−1−e−iθj3−1−1−13]=18−2cos⁡θj,L0=1,  L1=det⁡[3−1−10−13−eiθj−1−1−e−iθj3−10−1−13]=36−12cos⁡θj,F0′=−1,  F1′=det⁡[3−e−iθj−e−iθj3]=8.

Making use of the initial conditions, respectively, and solving (22), we obtain

(24) Fn=18−2cos⁡(2jπ/m)440−48cos⁡(2jπ/m)+8cos⁡2(2jπ/m)(an−bn);Ln=36−12cos⁡(2jπ/m)−b440−48cos⁡(2jπ/m)+8cos⁡2(2jπ/m)an−36−12cos⁡(2jπ/m)−a440−48cos⁡(2jπ/m)+8cos⁡2(2jπ/m)bn;Fn′=8+b440−48cos⁡(2jπ/m)+8cos⁡2(2jπ/m)an−8+a440−48cos⁡(2jπ/m)+8cos⁡2(2jπ/m)bn,

where $a=14-6\cos\left(2j\pi /m\right)+2\sqrt{40-48\cos\left(2j\pi /m\right)+8\cos^{2}\left(2j\pi /m\right)}$ and $b=14-6\cos(2j\pi /m)-2\sqrt{40-48\cos(2j\pi /m)+8\cos^{2}(2j\pi /m)}$.

By combining (19) and (24), we obtain

(25) ϕj(0)=a2n+b2n−2(6+2cos⁡(2jπm))2n,j=1,…,m2−1.

Similarly, by calculation, we have

(26) t(L4.8.80)=det⁡(−Am′−Cm′){1}=Fn|j=m=16Fn−1−64Fn−2=2n8n.

Expanding the determinant along the first row and then expanding the resulting determinants along the first column, we have

(27) det⁡[Am/2′+Cm/2′]=3Fn−3(6+2cos⁡π)Ln−1+(6+2cos⁡π)Fn−1−Fn′−2(6+2cos⁡π)n=an+bn−2(6+2cos⁡π)n=4n[(5+26)n+(5−26)n−2].

Thus, we have the following.

**Theorem 3 .**

The number of spanning trees of 4.8.8 lattice can be expressed as

(28) t(L4.8.8K)={2  ×  32nnm[(5+26)n+(5−26)n−2]   ×∏j=1m/2−1[a2n+b2n−2(6+2cos⁡⁡(2jπm))2n],(m  is  even) 2×8nnm×∏j=1(m−1)/2[a2n+b2n−2(6+2cos⁡⁡(2jπm))2n], (m  is  odd), 

where $a=14-6\cos\left(2j\pi /m\right)+2\sqrt{40-48\cos\left(2j\pi /m\right)+8\cos^{2}\left(2j\pi /m\right)}$ and $b=14-6\cos\left(2j\pi /m\right)-2\sqrt{40-48\cos\left(2j\pi /m\right)+8\cos^{2}\;\left(2j\pi /m\right)}$.

## 3. The Hexagonal Lattice

The hexagonal lattice *L*_*h*_ is shown in Figure 1(b). If we identify *a*_1_ and *b*_1_*, *a*_m_* and *b*_2n_*, and *b*_*s*_ and *b*_s_* for *f* or *s* = 2,…, 2*n* − 1 in *L*_*h*_, we obtain a graph with cylindrical boundary condition, denoted by *L*_*h*_^*c*^. Adding edges (*a*_*s*_, *a*_*m*+1−s_*) for 1 ≤ *s* ≤ *m*, in *L*_*h*_^*c*^, a hexagonal lattice with toroidal boundary condition, denoted by *L*_*h*_^*t*^, can be gotten.

Yan and Zhang [4] got the number of spanning trees and the asymptotic tree number entropy of *L*_*h*_^*c*^:

(29) t(Lhc)=2nm∏j=1m−1ab[(c+ab)n−(c−ab)n],z(Lhc)=lim⁡m,n→∞12mnlog⁡t(Lhc)=14π∫02πlog⁡⁡(3−cos⁡x+7−8cos⁡x+cos⁡2x)dx≈0.8077,

where *a* = 1 − cos⁡(2*jπ*/*m*), *b* = 7 − cos⁡(2*jπ*/*m*), and *c* = 3 − cos⁡(2*jπ*/*m*).

Shrock and Wu [7] showed that the number of spanning trees and the asymptotic tree number entropy of *L*_*h*_^*t*^ can be expressed as

(30) t(Lht)=3nm∏s=0n−1 ∏j=0m−1(s,j)≠(0,0)[6−2cos⁡θ1−2cos⁡θ2−2cos⁡(θ1+θ2)],z(Lht)=lim⁡m,n→∞12mnlog⁡t(Lht)=18π2∬02πlog⁡⁡(6−2cos⁡x−2cos⁡y⁡−2cos⁡(x+y))dx dy,≈0.8077,

where *θ*_1_ = 2*sπ*/*n* and *θ*_2_ = 2*jπ*/*m*.

By adding edges (*a*_*s*_, *a*_s_*) for 1 ≤ *s* ≤ *m*, in *L*_*h*_^*c*^, a hexagonal lattice *L*_*h*_^*K*^ with Klein bottle boundary condition can be gotten. For the number of spanning trees of *L*_*h*_^*K*^, we have the following result.

**Theorem 4 .**

The number of spanning trees of hexagonal lattice can be expressed as

(31) t(LhK)={3×24n−1nm ×∏j=1m/2−1[a2n+b2n−2n+1×(1+cos⁡⁡(2jπm))n],if  m  is  even,3×2n−1nm ×∏j=1(m−1)/2[a2n+b2n−2n+1×(1+cos⁡⁡(2jπm))n],if  m  is  odd,

where $a=3-\cos\left(2j\pi /m\right)+\sqrt{7-8\cos\left(2j\pi /m\right)+\cos^{2}\left(2j\pi /m\right)}$ and $b=3-\cos\left(2j\pi /m\right)-\sqrt{7-8\cos\left(2j\pi /m\right)+\cos^{2}\left(2j\pi /m\right)}$.

**Proof**

By suitable labelling of vertices of *L*_*h*_^*K*^, the adjacency matrix *X* of it can be written in terms of a linear combination of direct products of smaller ones:

(32) X=A⊗Im+B⊗K1+BT⊗K1T+C⊗K2,

where

(33) A=[A1InInA2],  B=[0n0nIn0n],C=[0n0n0nC1],

*A*_1_ = (*a*_*sj*_^1^)_*n*×*n*_, in which *a*_*sj*_^1^ = *a*_*js*_^1^ = 1 if *s* is odd and *j* = *s* + 1; else, *a*_*sj*_^1^ = 0; *A*_2_ = (*a*_*sj*_^2^)_*n*×*n*_, in which *a*_*sj*_^2^ = *a*_*js*_^2^ = 1, if *s* is even, and *j* = *s* + 1; else, *a*_*sj*_^2^ = 0; *C*_1_ = (*c*_*sj*_)_*n*×*n*_, in which *c*_1*n*_ = *c*_*n*1_ = 1, otherwise 0.

Interchanging rows and columns, we have

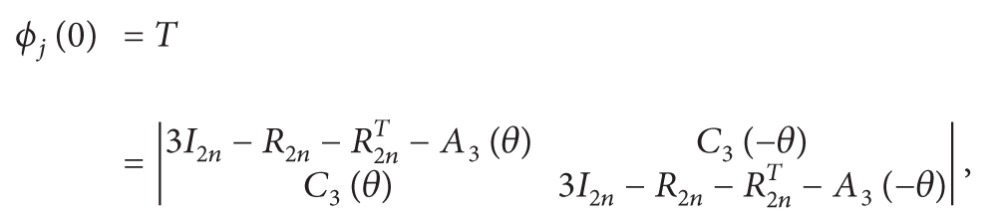
(34)
where *A*_3_(*x*) = (*a*_*sj*_^3^)_2*n*×2*n*_, in which *a*_*sj*_^3^ = *a*_*j*+2,*s*+2_^3^ = *e* ^(−1)^*i*/2^*x*^, if *s* is even, and *j* = *s* + 1, *a*_2*n*−1,2*n*_^3^ = 1 + *e* ^−*ix*^, and *a*_2*n*,2*n*−1_^3^ = 1 + *e* ^*ix*^ *; else, a*_*sj*_^3^ = 0; *C*_3_(*x*) = (*c*_*sj*_^3^)_2*n*×2*n*_, in which *c*_1,2*n*_^3^ = −1 − *e* ^*ix*^, *c*_2*n*,1_^3^ = −1 − *e* ^−*ix*^, otherwise 0. Expanding the determinant *T* along the first row and then expanding the resulting determinants along the first column, we have

(35) T=3T{1}−T{1,2,3}−(2+2cos⁡θj)T{1,4n}−2×152n(2+2cos⁡θj)n.

Let *F*_4*n*−1_ = *T* ^{1}^, *L*_2(2*n*−1)_ = *T* ^{1,2}^, *F*_4*n*−2_′ = *T* ^{1,4*n*}^, and *L*_2(2*n*−1)_′ = *T* ^{1,2,4*n*}^. Also, set Γ_*j*−1_ = Γ_*j*_^{1,2}^, *j* = 2,…, *n*, Γ_*n*_ ∈ {*F*_*n*_, *L*_*n*_, *F*_*n*_′, *L*_*n*_′}.

By the Laplace expansion theorem, we obtain several expansions. First, an expansion by rows 1 and 2 is as follows:

(36) $$\begin{array}{c} F_{n}=3L_{n-1}-\left(2+2\cos\theta_{j}\right)F_{n-2};\\ F_{n}^{\prime }=3L_{n-1}^{\prime }-\left(2+2\cos\theta_{j}\right)F_{n-2}^{\prime }. \end{array}$$

An expansion by rows 1 and 2 is as follows:

(37) $$\begin{array}{c} L_{n}=3L_{n-1}-F_{n-1};\\ L_{n}^{\prime }=3L_{n-1}^{\prime }-F_{n-1}^{\prime }. \end{array}$$

The recursion relations (36) and (37) give

(38) Γn=(6−2cos⁡θj)Γn−1−(2cos⁡θj+2)Γn−2, Γn∈{Fn,Ln,Fn′,Ln′}.

Note that

(39) F3=det⁡[3−1−eiθj0−1−eiθj3−eiθj0−eiθj3]=18−6cos⁡θj, F1=3,L2=det⁡[3−eiθj−e−iθj3]=8, L0=1,F2′=det⁡[3−1−eiθj−1−e−iθj3]=7−2cos⁡θj, F0′=1.

Making use of the initial conditions, respectively, and solving (38), we obtain

(40) Fn=9−3cos⁡⁡(2jπ/m)+3c2ca(n−1)/2−9−3cos⁡⁡(2jπ/m)−3c2cb(n−1)/2;Ln=5+cos⁡(2jπ/m)+c2can/2−5+cos⁡(2jπ/m)−c2cbn/2;Fn′=4−cos⁡(2jπ/m)+c2can/2−4−cos⁡⁡(2jπ/m)−c2cbn/2,

where *a* = 3 − cos⁡(2*jπ*/*m*) + *c*, *b* = 3 − cos⁡(2*jπ*/*m*) − *c* and $c=\sqrt{7-8\cos(2j\pi /m)+\cos^{2}(2j\pi /m)}$. By combining (35) and (40) we obtain

(41) ϕj(0)=a2n+b2n−2n+1152n(1+cos⁡(2jπm))n,j=1,…,m2−1.

Similarly, by calculation, we have

(42) t(Lh0)=ϕm(0){1}=det⁡(−Am′−Cm′){1}=F2n−1|j=m=3n2n−1.

Expanding the determinant along the first row and then expanding the resulting determinants along the first column, we have

(43) det⁡[Am/2′+Cm/2′]=3F2n−1−L2n−2 −(2+2cos⁡π)F2n−1′−2×152n ×(2+2cos⁡π)n=an+bn−2 ×152n(2+2cos⁡π)n=8n.

By formula (18), the result holds.

## 4. The 3^3^ · 4^2^ Lattice

The 3^3^ · 4^2^ lattice can be constructed by starting with the square lattice and adding a diagonal edge connecting the vertices in the upper left to the lower right corners of each square in every other row as shown in Figure 1(c). If we identify *a*_1_ and *b*_1_*, *a*_m_* and *b*_2n_*, and *b*_*s*_ and *b*_s_* for *s* = 2,…, 2*n* − 1, in *L*_3^3^·4^2^_, we obtain a graph with cylindrical boundary condition, denoted by *L*_3^3^·4^2^_^*c*^. Adding edges (*a*_*s*_, *a*_*m*+1−s_*) for 1 ≤ *s* ≤ *m*, in *L*_3^3^·4^2^_^*c*^, a 3^3^ · 4^2^ lattice with toroidal boundary condition, denoted by *L*_3^3^·4^2^_^*t*^, can be gotten.

Yan and Zhang [4] got the number of spanning trees and the asymptotic tree number entropy of *L*_3^3^·4^2^_^*c*^:

(44) t(L33·42c)=2nm∏j=1m−1ab[(c+ab)n−(c−ab)n],z(L33·42c)=lim⁡m,n→∞12mnlog⁡⁡t(L33·42c)=14π∫02πlog⁡[11−11cos⁡x+2cos⁡2x+((11−11cos⁡x+2cos⁡2x)2−2−2cos⁡x)1/2]dx≈1.4069,

where *a* = 7 − 9cos⁡(2*jπ*/*m*) + 2cos⁡^2^(2*jπ*/*m*), *b* = 17 − 13cos⁡(2*jπ*/*m*) + 2cos⁡^2^(2*jπ*/*m*), and *c* = 11 − 11cos⁡(2*jπ*/*m*) + 2cos⁡^2^(2*jπ*/*m*).

Chang and Wang [9] showed that the number of spanning trees and the asymptotic tree number entropy of *L*_3^3^·4^2^_^*t*^ can be expressed as

(45) t(L33·42t)=3nm∏s=0m−1 ‍∏j=0n−1(s,j)≠(0,0)[22−22cos⁡θ1+4cos⁡2θ1−2cos⁡θ2−2cos⁡⁡(θ1−θ2)],z(L33·42t)=lim⁡m,n→∞12mnlog⁡t(L33·42t)=14π∫02πlog⁡[11−11cos⁡x+2cos⁡2x+((11−11cos⁡x+2cos⁡2x)2,−2−2cos⁡x)1/2]dx≈1.4069,

where *θ*_1_ = 2*sπ*/*m* and *θ*_2_ = 2*jπ*/*n*.

By adding edges (*a*_*s*_, *a*_s_*) for 1 ≤ *s* ≤ *m*, in *L*_3^3^·4^2^_^*c*^, a 3^3^ · 4^2^ lattice *L*_3^3^·4^2^_^*K*^ with Klein bottle boundary condition can be gotten. For the number of spanning trees of *L*_3^3^·4^2^_^*K*^, we have the following theorem.

**Theorem 5 .**

The number of spanning trees of 3^3^ · 4^2^ lattice *L*_3^3^·4^2^_^*K*^ can be expressed as

(46) t(L33.42K)={3×2n−1×48nnm ×∏j=1m/2−1[a2n+b2n−2 ×(2+2cos⁡(2jπm))n],if  m  is  even,3×2n−1nm ×∏j=1(m−1)/2[a2n+b2n−2 ×(2+2cos⁡(2jπm))n],if  m  is  odd,

where $a=\left[11-11\cos\left(2j\pi /m\right)+2\cos^{2}\left(2j\pi /m\right)\right]+\sqrt{{\left[11-11\cos\left(2j\pi /m\right)+2\cos^{2}\left(2j\pi /m\right)\right]}^{2}-2-2\cos\left(2j\pi /m\right)}$ and $b=[11-11\cos\left(2j\pi /m\right)+2\cos^{2}\left(2j\pi /m\right)]-\sqrt{{\left[11-11\cos\left(2j\pi /m\right)+2\cos^{2}\left(2j\pi /m\right)\right]}^{2}-2-2\cos\left(2j\pi /m\right)}$.

**Proof**

By a suitable lebelling of vertices of *L*_3^3^·4^2^_^*K*^, the adjacency matrix *X* of it can be written in terms of a linear combination of direct products of smaller ones:

(47) X=A⊗Im+B⊗K1+BT⊗K1T+C⊗K2,

where *A* = *R*_2*n*_ + *R*_2*n*_^*T*^ and *B* = (*b*_*sj*_)_2*n*×2*n*_, where *b*_*sj*_ = 1, if *s* is odd, and *j* = *s* + 1; else, *b*_*sj*_ = *δ*_*sj*_, *C* = (*c*_*sj*_)_2*n*×2*n*_, in which *c*_1,2*n*_ = *c*_2*n*,1_ = 1, otherwise 0. Using the same notations as Section 2, we have

(48) ϕj(0)=(5−2cos⁡θj)ϕj(0){1}−(2+2cos⁡θj)ϕj(0){1,2} −ϕj(0){1,12n}−2×(2+2cos⁡θj)n.

Let *F*_2*n*_ = *ϕ*_*j*_(0)^{1}^, *L*_2*n*−1_ = *ϕ*_*j*_(0)^{1,2}^, *F*_2*n*−1_′ = *ϕ*_*j*_(0)^{1,2*n*}^, and *L*_2*n*−1_′ = *ϕ*_*j*_(0)^{1,2,2*n*}^. Also set Γ_*j*−1_ = Γ_*j*_^{1,2}^, *j* = 2,…, *n*, Γ_*n*_ ∈ {*F*_*n*_, *L*_*n*_, *F*_*n*_′, *L*_*n*_′}.

By the Laplace expansion theorem, we obtain several expansions. First, an expansion by rows 1 and 2 is as follows:

(49) $$\begin{array}{c} F_{n}=\left(5-2\cos\theta_{j}\right)L_{n-1}-F_{n-1};\\ F_{n}^{\prime }=\left(5-2\cos\theta_{j}\right){L_{n-1}^{\prime }}_{}-F_{n-1}^{\prime }. \end{array}$$

An expansion by rows 1 and 2 is as follows:

(50) $$\begin{array}{c} L_{n}=\left(5-2\cos\theta_{j}\right)L_{n-1}-\left(2+2\cos\theta_{j}\right)F_{n-1};\\ L_{n}^{\prime }=\left(5-2\cos\theta_{j}\right)L_{n-1}^{\prime }-\left(2+2\cos\theta_{j}\right)F_{n-1}^{\prime }. \end{array}$$

The recursion relations (49) and (50) give

(51) Γn=(22−22cos⁡(2jπm)+2cos⁡2(2jπm))Γn−1−(2cos⁡θj+2)Γn−2, Γn∈{Fn,Ln,Fn′,Ln′}.

Note that

(52) F0=0,  F1=5−2cos⁡θj,L0=1,L1=det⁡[5−2cos⁡θj−1−eiθj−1−e−iθj5−2cos⁡θj]=23−22cos⁡θj+4cos⁡2θj,F0′=1,F1′=det⁡[5−2cos⁡θj−1−15−2cos⁡θj]=24−20cos⁡θj+4cos⁡2θj.

Making use of the initial conditions, respectively, and solving (51), we obtain

(53) Fn=5−2cos⁡(2jπ/m)c(an−bn);Ln=23−22cos⁡θj+2cos⁡2θj−bcan−23−22cos⁡θj+2cos⁡2θj−acbn;Fn′=24−20cos⁡θj+2cos⁡2θj+bcan−24−20cos⁡θj+2cos⁡2θj+acbn,

where $a=\left(11-11\cos\left(2j\pi /m\right)+2\cos\left(2j\pi /m\right)\right)+\sqrt{c},\;b=(11-11\cos(2j\pi /m)+2\cos(2j\pi /m))-\sqrt{c}$,  and *c* = (11 − 11cos⁡(2*jπ*/*m*) + 2cos⁡(2*jπ*/*m*))^2^ − 2 − 2cos⁡(2*jπ*/*m*). By combining (48) and (53) we obtain

(54) ϕj(0)=a2n+b2n−2×(2+2cos⁡(2jπm))n, j=1,…,m2−1.

Similarly, by calculation, we have

(55) t(L33·420)=ϕm(0){1}=det⁡(−Am′−Cm′){1}=3×  2n−1n.

When *m* is even, expanding the determinant along the first row and then expanding the resulting determinants along the first column, we have

(56) det⁡[Am/2′+Cm/2′]=(5−2cos⁡π)Fn−(2+2cos⁡π)Ln−1 −Fn−1′−2×(1+cos⁡π)n=an+bn−2×(1+cos⁡π)n=48n.

By formula (18), the result holds.

## 5. Concluding Remarks

In this paper, we computed the numbers of spanning trees for 4.8.8 lattice, hexagonal lattice, and 3^3^ · 4^2^ lattice with a Klein bottle boundary condition. For the asymptotic tree number entropy of graphs, Lyons [11] got the following result.

**Theorem 6 .**

Let {*G*_*n*_} be a tight sequence of finite connected graphs with bounded average degree such that lim⁡_*n*→*∞*_(log⁡*t*(*G*_*n*_)/|*V*(*G*_*n*_)|) = *h*. If {*G*_*n*_′} is a sequence of connected subgraph of {*G*_*n*_}, such that lim⁡_*n*→*∞*_(|{*v* ∈ *V*(*G*_*n*_′); *d*_*G*_*n*__(*v*) = *d*_*G*_*n*_′_(*v*)}|/|*V*(*G*_*n*_)|) = 1, then lim⁡_*n*→*∞*_(log⁡*t*(*G*_*n*_′)/|*V*(*G*_*n*_′)|) = *h*.

By Theorem 6 (or compared with the results by Chang and Shrock [8], Chang and Wang [9], Shrock and Wu [7], and Yan and Zhang [4]), we can see that 4.8.8 lattices have the same asymptotic tree number entropy with three different boundary conditions (cylindrical, toroidal, and Klein bottle). Also hexagonal lattice and 3^3^ · 4^2^ lattice have the same property.
